# Clinical Assessment of the Hyperbaric Oxygen Therapy Efficacy in Mild to Moderate Periodontal Affections: A Simple Randomised Trial

**DOI:** 10.3390/medicina58020234

**Published:** 2022-02-04

**Authors:** Alexandru Burcea, Laurenta Lelia Mihai, Anamaria Bechir, Mircea Suciu, Edwin Sever Bechir

**Affiliations:** 1 Faculty of Dental Medicine, Titu Maiorescu University of Bucharest, 031593 Bucharest, Romania; alexandru.burcea@helpdent.ro; 2 Faculty of Dental Medicine, George Emil Palade University of Medicine, Pharmacy, Science and Technology of Targu Mures, 540142 Targu Mures, Romania; oralmed2000@yahoo.com (M.S.); bechir.edwin@gmail.com (E.S.B.)

**Keywords:** periodontal disease, oral health index, dental mobility, periodontal pockets depth, hyperbaric oxygen therapy

## Abstract

*Background and Objectives:* Gum disease represents the condition due to the dental plaque and dental calculus deposition on the surfaces of the teeth, followed by ulterior destruction of the periodontal tissues through the host reaction to the pathogenic microorganisms. The aim of study was to present aspects regarding the efficacy of hyperbaric oxygen therapy (HBOT) as an adjuvant therapy for the treatment of periodontal disease, started from the already certified benefits of HBOT in the general medicine specialties. *Materials and Methods:* The participant patients in this study (71) required and benefited from specific periodontal disease treatments. All patients included in the trial benefited from the conventional therapy of full-mouth scaling and root planing (SRP) within 24 h. HBOT was performed on the patients of the first group (31), in 20 sessions, of one hour. The patients of the control group (40) did not benefit from HBO therapy. *Results:* At the end of study, the included patients in HBOT group presented significantly better values of oral health index (OHI-S), sulcus bleeding index (SBI), dental mobility (DM), and periodontal pocket depth (PD) than the patients of the control group. *Conclusions:* HBOT had beneficial effects on the oral and general health of all patients, because in addition to the positive results in periodontal therapy, some individual symptoms of the patients diminished or disappeared upon completion of this adjuvant therapy.

## 1. Introduction

Dental biofilm is the main etiologic factor for caries, periodontal and peri-implant infections. Gum disease represents a disease with dental plaque and calculus formation on the surfaces of the teeth, followed by ulterior destruction of the periodontal tissues due to the host reaction to pathogenic microorganisms [1,2].

The spreading of periodontal disease is between 20 and 50% in the world, and represents one of important causes of indentations, which jeopardize the functions of oro-facial system, including mastication, aesthetics, self-reliability, and life quality [3]. This prevalence of periodontal disease is presumed to be rising in future years because of increased aging in population and of maintenance of natural teeth of dental arches in the elderly [4,5]. The development of periodontal disease in the context of the 2017 World Workshop on the Classification of Periodontal and Peri-Implant Diseases and Conditions presents four stages, defined based on severity (primarily periodontal breakdown with reference to root length and periodontitis-associated tooth loss), complexity of management (pocket depth, infrabony defects, furcation involvement, tooth hypermobility, masticatory dysfunction) and additionally described as extent (localized or generalized). The grade of periodontitis is estimated with direct or indirect evidence of progression rate in three categories: slow, moderate and rapid progression (Grade A–C). Risk factor analysis is used as grade modifier [6,7].

Cellular oxygenation is carried out by transporting the oxygen (that is fixed to haemoglobin), from inspired air to the tissues and cells, through the circulatory system. Disruption or interruption of oxygen transport induces hypoxia through changes in haemoglobin, capillary network, or blood flow [8,9,10]. Oxygen has a very small molecule, which allows its increased diffusibility in tissues, in comparison to any other substance [11]. Increasing the oxygen pressure in the environment over a certain point, increases the amount of dissolved oxygen in the plasma (quantitative increase) and the penetration rate of oxygen into the tissues (qualitative increase) [12].

The Undersea and Hyperbaric Medical Society (UHMS) defines hyperbaric oxygen therapy (HBOT) as a treatment in which the patient intermittently inspires 100% oxygen, in a pressurized treatment chamber at a higher pressure than at the sea level (1 atm absolute, ATA) [11,13,14]. In HBO therapy, the patient inspires pure or enriched oxygen, which causes a reduction in the amount of nitrogen in the blood [15]. The mechanisms of therapeutic action of HBOT are based on raising the partial pressure of inspired oxygen and increasing hydrostatic pressure, by compressing the gas in all spaces in the body, according to Boyle’s law [16,17]. Increasing the oxygen’s partial pressure raises its diffusibility in tissues. Even if there is a similar amount of oxygen in the plasma and in the transported oxygen by haemoglobin, by increasing the oxygen’s partial pressure, its effectiveness is enhanced at a cellular level [18]. Increasing the pressure of the oxygen in the environment over a certain point induces the rising of the amount of oxygen dissolved in the plasma (increase in volume), and the penetration of the oxygen in the tissues (increase in quality) [19,20]. Therefore, HBO treatment leads to a considerable development in bone formation, so that the lamellar bone grows [21].

In the study effectuated by Giacon et al. [22], the authors consider that through HBO therapy pre-treatment, the protection proteins of oxidative stress are activated and tissues are prepared for surgery entailing transient ischemia. They presented a case report of an immediate dental implant, which depicted the utilisation of HBO therapy and of advanced platelet-rich fibrin (A-PRF) for pre-treating the implant site in a case of severe periodontitis with tooth attachment loss. Three sessions of HBO therapy effectuated for preconditioning by increasing the positive results, presumably through sterilization of surgical site and development of antioxidant protection. The authors underlined that future studies should specifically address this topic.

Altug et al. [23], studied the consequences of HBO therapy on implant osseointegration in experimental diabetes rabbits. The authors concluded that the disclosures in histomorphometry hint that HBO therapy has a certain impact on the osseointegration of dental implants, in the early healing time in diabetic rabbits. The authors underlined that dental implant stability is not influenced by HBO therapy.

The administration of oxygen in HBOT is realized in hyperbaric chambers, which can be multiplace (type A), and monoplace (type B, that provides treatment for a single patient) [24]. Both chambers are used to treat various diseases, from simple injuries to serious illness [25].

The comparative study started from the already certified benefits of HBOT in the general medicine specialties. The aim of this comparative study was to find out if the adjuvant HBO therapy presents beneficial effects in the treatment of adult patients affected by periodontal disease, after the conventional therapy of performing professional dental hygienization measures, and of full-mouth scaling and root planing (SRP) with EMS Piezon device and EMS Air-Flow Master. The expected result of the trial should potentially contribute to an advanced treatment strategy for periodontal disease, with an ideal clinical outcome.

## 2. Materials and Methods

The study was realized in conformity to the ethical principles of the Declaration of Helsinki and of the good clinical practice. The protocol was approved by the Ethics Committee of Dental Medicine Faculty, Titu Maiorescu University of Bucharest (No. 2 of 5 May 2017). All patients were informed about the research requirements, attended only by those that entered voluntarily in the research program. The study phases were explained to each recruited patient, including the need for monitoring. Included patients signed the written informed consent prior to the beginning of this study. The working hypothesis started from the premise that the benefits of HBOT are already certified in general medicine specialties, but can it be used as an adjuvant therapy in Romanian patients with periodontal disease?

The study was conducted in the Clinics of Dental Medicine Faculties, between of May 2017 and May 2021, but the COVID-19 pandemic epidemiological context determined a 12-month intermission. The authors followed calibration trainings to ensure: the precision and correctness of patients’ anamneses, of clinical examination and diagnosis; the proper use of EMS Piezon and EMS Air-Flow Master Device; the use of the same standardized clinical measurements. The calibration trainings were realized in order to ensure the validity and reliability of clinical study and of obtained results. Adjuvant HBO therapy was performed in Hypermed Care SRL Clinic, by using the Revitalair^®^430 monoplace equipment, produced by Biobarica (Medley, FL, USA).

**Figure 1:**
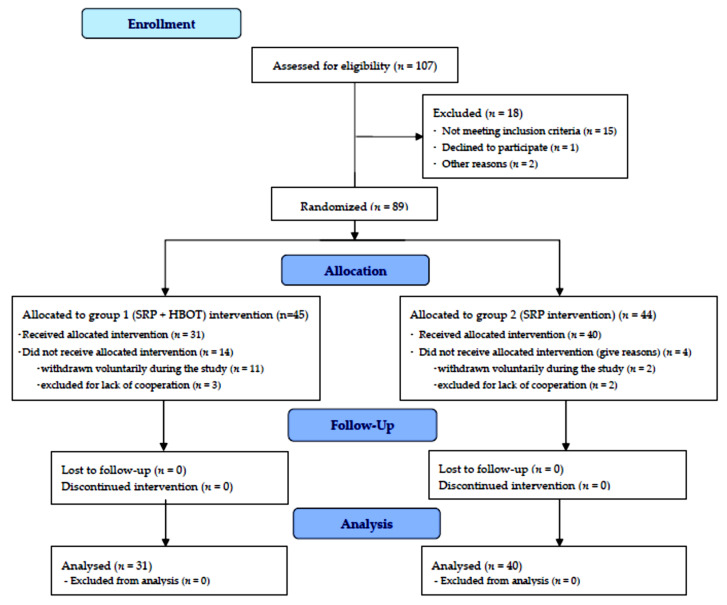
Flow Diagram of the study.

Clinical examinations and interviews were accomplished to evaluate eligibility. Patients were randomly screened, and then asked to participate in the study. Study initially enrolled 89 patients, but 12 subjects withdrawn voluntarily during the study and 6 subjects were excluded for lack of cooperation. The remaining patients in the study (71), had the age range of 38–59 years (means 48.5 years, ±10.5 years). Table 1 presents the sample patients and Figure 1 presents the flow diagram of the study.

Oral examinations and X-rays were effectuated at the time of patients selection, to differentiate the periodontal affection from other diseases (e.g., apical periodontitis, tooth fracture, etc.). The oral examinations consisted of the assessment of periodontal status by determination of the oral hygiene conditions and of Simplified Oral Health Index (OHI-S), Sulcus Bleeding Index (SBI), examination of clinical attachment level (CAL), checking for dental mobility degree (MD), of the pocket depth (PD), and of furcation involvement. All periodontal examinations were effectuated by using mirrors, tweezers, probes, and a calibrated periodontal UNC-PCP15 Color-Coded Probe (Hu-Friedy Europe, The Netherlands) probe.

Inclusion criteria for this study consisted of non-smoking patients aged 38–59 years old, having at least 6 natural teeth on a half dental arch (excluding third molars), having periodontal symptomatology at the presentation in the dental office (like gum redness or/and bleeding, gum swelling, persistent metallic taste, halitosis, painful chewing, sensitive teeth, minimum 4 teeth with first or second degree of dental mobility, periodontal pockets), or with a confirmed diagnosis of mild to moderate periodontal disease.

All selected patients for admission into the first group of patients with HBO therapy completed and signed a fact sheet with information regarding their general and specific health conditions (which contained affections which determined the exclusion criteria of study). Inclusion criteria of patients are depicted in Table 2.

Exclusion criteria for this study were represented by smoker patients, maximum scores of all studied periodontal indices, periodontal treatment and antibiotic therapy in the last six months, aggressive periodontitis, endodontic affections, orthodontic patients, patients with infections, systemic disorders, upper respiratory and pulmonary disorders (e.g., untreated pneumothorax, pneumonia, asthma, chronic obstructive pulmonary disease, etc.), cataract, Eustachian tube dysfunction, hereditary spherocytosis, fever, claustrophobia, convulsions, cardiac simulators or other implanted or external devices that control body functions, patients with unstable cardiovascular disease, pregnant woman, hospitalized patients, those with cancer, with uncontrolled diabetes mellitus, with parafunction of chewing habit, with severe malocclusion, those with missing data, patients with mental disability, uncooperative patients, and patients who refused to be included in the study.

According to the safety requirements of patients, those with general medical conditions presented in exclusion criteria of patients cannot be accepted for HBO therapy, thus patients with these affections were not be admitted in study group 1 (with HBOT). Exclusion criteria are presented in Table 3.

Supra- and subgingival debridement, scaling, and root planing with EMS Piezon device and EMS Air-Flow Master device, followed by manual root planing were effectuated in every selected patient in 24 h. Guided Biofilm Therapy with EMS device is a treatment protocol built in conformity of each patient diagnosis and risk evaluation for achieving optimum outcomes. The treatment is realized in a minimally invasive way, with the highest comfort, safety and efficiency for the patients. Oral hygiene instructions were presented to every participant patient, and dental plaque disclosing gel (GC Tri Plaque ID Gel) was used before any assessment sessions. Patients included in the study used the same toothpaste (Colgate Total Gum Protection Toothpaste), same dental brush (Colgate Gum Health Extra Soft Toothbrush for Sensitive Gums with Deep Cleaning Floss-Tip Bristles), and same interdental pick (GUM-6326RA Soft-Picks Original Dental Picks, small). Participants brushed their teeth twice a day, morning and evening, at least two minutes each time (in conformity with the American Dental Association (ADA) suggestions), with Stillman method of tooth brushing. An amount of 1 cm tooth paste was used. The inter-dental pick was indicated to be utilized only in the evening, before teeth brushing. Two tubes of plaque revealing gel were handed to the patients participating in the study, for checking their dental hygiene 3 times weekly. The gel was applied after tooth brushing, and if the sanitization was not correct, the patients had to perform their tooth brushing again. At the end of the study, the used quantity of the plaque disclosing gel from the tubes was verified, in order to verify the compliance of participants to the study protocol.

The selected patients (71), were divided into two groups, the first group of patients (group I) who agreed upon and benefited of HBOT adjuvant therapy (31 patients, 15 women and 16 men), and the second group of patients (group II / control group), who did not undergo HBO therapy (40 patients, 19 women and 21 males). Both patient groups were selected according to the same inclusion/exclusion criteria and benefited from the same dental treatment protocol for periodontal disease, excepting HBO adjuvant therapy (which was realized only in 1st group of patients). The type of study design was simple randomized trial, by centralized randomization into groups, than participant patients were divided into the two groups by centralized randomization.

**Figure 2:**
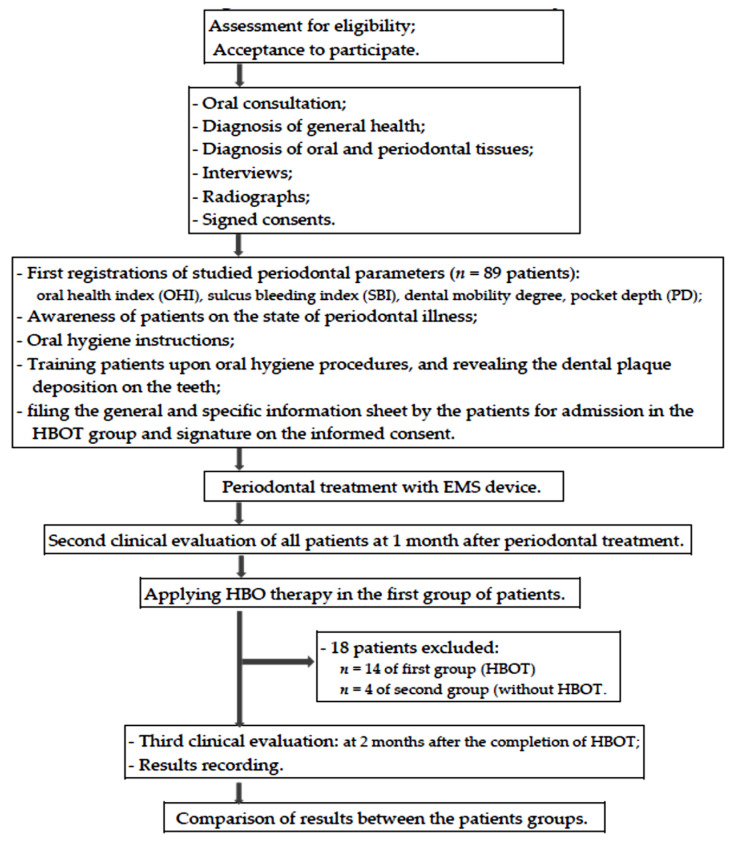
Flow chart of the clinical study.

The applied clinical protocol to all patients consisted of: consultation and complementary radiographic examinations; diagnosis of general health and of oral/periodontal tissues; first clinical evaluation and registration of dental and periodontal status by determination of OHI-S index, of the sulcus bleeding index, the pathological tooth mobility and the depth of periodontal pockets; establishing of the treatment plan; filing the general and specific information sheet (by the patient for admission in the HBOT group), and signature on the informed consent; awareness of patients on the state of periodontal illness at presentation; training and insisting upon artificial oral hygiene procedures by using the Stillman technique of tooth brushing; taking of the same brand and type of toothbrushes, toothpastes, interdental brushes, and dental plaque disclosing gel; conducting of the corrective treatment (performing professional oral hygiene, and of specific therapy for dental and periodontal diseases with EMS devices); second clinical evaluation of the patients at 1 month after periodontal treatment; applying HBO therapy in the first group of patients; third clinical evaluation at 2 months after the completion of HBO therapy and recording of the results; comparison of results. Figure 2 depict the flow chart of the clinical study.

**Figure 3:**
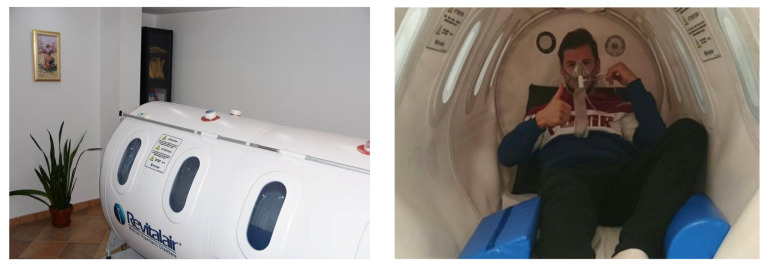
The monoplace hyperbaric chamber used in Biobarica Hypermed Care Clinic.

After the effectuation of specific periodontal treatment in all patients, each patient belonging to the first group (with HBO adjuvant therapy) was set to perform 20 sessions of hyperbaric oxygen therapy for one hour, with the applied pressure of 1.4 ATM. The frequency of the therapy sessions was three sessions per week. HBO therapy was performed at the Biobarica Hypermed Care Clinic in Bucharest, in a monoplace hyperbaric chamber (Figure 3).

Stillman method of tooth brushing consists of thoroughly brushing around and under the gum line, and it helps to clean the debris deposited between the teeth, because the toothbrush bristles reach under the gums [25].

Oral hygiene status can be determined by using the oral health index (OHI), calculated by the oral debris score and the dental calculus score found on the buccal and lingual surfaces of each of the three segments of the dental arches. The calculation of the numerical values of simplified oral health index (OHI-S) is in conformity to the existing dental plaque and calculus deposits. The simplified oral hygiene index (OHI-S) allows the separate evaluation of the soft and hard deposits, present on the buccal/labial or oral dental crown surfaces of 6 teeth of both dental arches, one tooth for each sextant: maxillary dental arch, teeth 1.6, 1.1, 2.6 on buccal/labial surfaces, respectively mandibular dental arch teeth 3.6, 3.1, 4.6 on lingual surfaces (enumerated teeth are noted after FDI notation system). The selected surfaces used for scoring were the buccal for maxillary molar and the lingual for mandibular molars, respectively, the labial for the maxillary right central incisors and for the mandibular left central incisors. In the absence of first molar, the second or third molar were examined, and in the case of the incisors, the neighbouring incisor.

The quantification of dental deposits can be performed visually or by staining solution. The surfaces of dental crowns are examined with the probe, extending the examination to the level of contact points of the proximal coronary surfaces, including the subgingival area. Debris index-simplified (DI-S) calculation method: 0 = absence of dental plaque; 1 = microbial plaque present up to 1/3 of the tooth surface; 2 = microbial plaque present between 1/3 and 2/3 of the tooth surface; 3 = microbial plaque present over 2/3 of the tooth surface. Calculus index-simplified (CI-S) calculation method: 0 = absence of dental calculus; 1 = calculus present up to 1/3 of the tooth surface; 2 = calculus present between 1/3 and 2/3 of the tooth surface; 3 = calculus present over 2/3 of the tooth surface. The averages for the plaque and tartar indices will be calculated and then added together; their sum will represent the OHI-S index [26,27]. OHI-S score is summed and then divided to the number of examined dental crown surfaces, for the mean oral hygiene score [28,29]. An arithmetic mean of the individual scores for debris and calculus index was performed, and subsequently the highest determined score was taken into consideration. The values of OHI-S index, necessary for interpretation, are: excellent 0; good 0.1–1.2; satisfactory 1.3–3.0; and unsatisfactory 3.1–6 [30]. Index interpretation of OHI-S scores used in this study was: excellent = 0; good = 1; satisfactory = 2; and unsatisfactory = 3. The scores were calculated according to the results of the determinations performed on each patient. An arithmetic mean of the individual scores was performed on each tooth, and subsequently the highest determined final score was taken into consideration.

Sulcus Bleeding Index (SBI, Műhlemann and Son) on gentle probing of the sulcus represents one of the initial signs of periodontal disease. In SBI, four gingival units are scored systematically for each tooth: the labial and lingual marginal gingival (M units) and the mesial and distal gingival papilla (P units). After probing, the examiner should wait for 30 s for scoring. Scores for these units are summed and then distributed to 4. Adding the obtained scores of the studied teeth and dividing them by the number of studied teeth establishes the sulcus bleeding index (SBI). Criteria of SBI scoring are: 0—healthy aspect of papilla and of marginal gingiva, without bleeding on probing; 1—healthy gingival aspect, but bleeding on probing; 2—bleeding on probing, modified colour, without edema; 3—bleeding on probing, modified colour, slight edema; 4—bleeding on probing, modified colour, evident edema; 5—spontaneous bleeding, modified colour, pronounced edema. SBI scoring is effectuated on the eight upper and lower anterior teeth, and four gingival areas are included for each tooth: mesial-labial, mesial-lingual, oro-mesial, oro-distal [31]. Index interpretation of BSI scores in this study was: excellent = 0; very good = 1; good = 2; satisfactory = 3; and unsatisfactory = 4.

The clinical sign of dental/tooth mobility depicts the periodontal destruction degree determined by local affections of gums and surrounding structures of the teeth. Tooth mobility presents 4 degrees: grade 0 represents the physiological mobility; in grade 1, the teeth present more than 1 mm mobility in a buccal-oral direction: in grade 2, the teeth present more than 1 mm mobility in buccal-oral and mesial-distal direction; and in grade 3, the teeth present mobility in three directions, buccal-oral, mesial-distal and incisal/occlusal-apical direction. Tooth mobility can represent a possible aggravation factor of the establishment of periodontal disease [32].

Periodontal pockets represent a pathological feature characterized by the displacement of the gingival attachment, respectively, the deepening of the gingival sulcus apically, due to the expansion of dental plaque and dental calculus towards the dental root. It can be classified as supra-alveolar (when the bottom of the pocket is situated at the crown of the alveolar bone), and intra-alveolar (when the bottom of the periodontal pocket is situated apical to the alveolar bone). Periodontal pockets can imply one or more tooth surfaces, and they can present various depths on different surfaces of the tooth. Periodontal pocket depth (PD) is a primary sign of periodontitis. The size and severity can be divided into normal (1 to 3 mm), early/mild periodontitis (4 to 5 mm), moderate periodontitis (5 to 7 mm), and severe periodontitis (7 to 12 mm). Periodontal examinations are effectuated with a periodontal probe [33]. In this study, an arithmetic mean of the individual scores for pockets was performed on each tooth, and subsequently the highest determined score was taken into consideration. Rationale of classifying periodontal pockets is to realize a correct evaluation, and then a correct prognosis of periodontal disease after the stage and grade of the disease, including the contributory factors, and after that, to effectuate the adequate treatment management of disease [6].

All the statistical analysis were performed in SPSS 24 Software. The considered level of significance is 0.05, otherwise mentioned. Data was analysed through means of the Chi-Square test for group differences.

## 3. Results

Two months after the end of HBO therapy in the first group of patients, we summarized and compared the recorded data of all patients. The obtained results in both studied groups, according to the three clinical examinations, are presented in Table 4.

**Figure 4:**
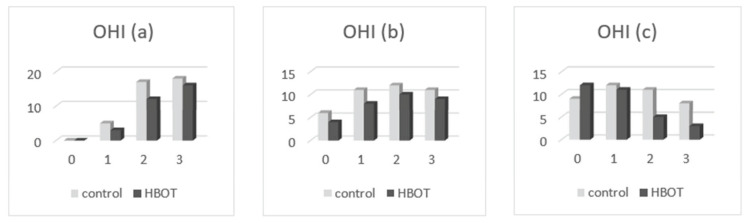
Patient distribution based on OHI index at (**a**) first examination, (**b**) second examination, and (**c**) third examination.

**Figure 5:**
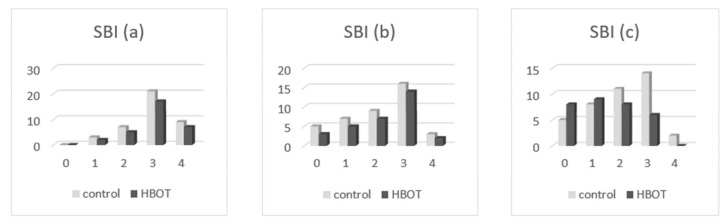
Patient distribution based on SB index at (**a**) first examination, (**b**) second examination, and (**c**) third examination.

**Figure 6:**
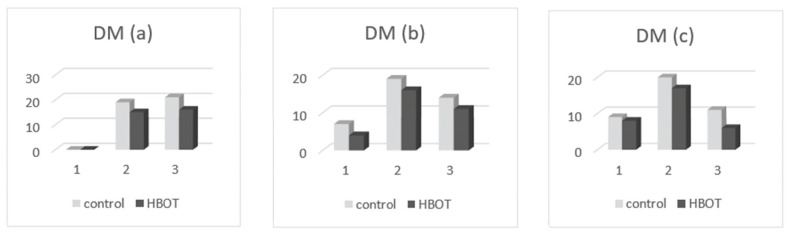
Patient distribution based on DM index at (**a**) first examination, (**b**) second examination, and (**c**) third examination.

**Figure 7:**
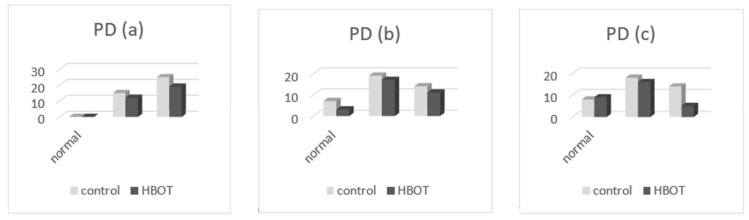
Patient distribution based on PD at (**a**) first examination, (**b**) second examination, and (**c**) third examination.

By comparing the listed values in Table 4, we found the following:

- Oral health index (OHI-S): at the presentation, the patients of both groups had only values of 1, 2, and 3 in OHI-S score, without value 0; in the second clinical examination, we found that OHI-S scores were reduced in both study groups (HBOT and control), because of SRP treatment, but probably also as a result of patient awareness in the need for correct oral hygiene through brushing exercises (initially done at the clinic, until all patients have acquired the correct technique), respectively, by revealing the bacterial plaque (performed at each presentation of the patient in the practice); at the second and third determination, we found that the OHI-S decreased in the patients of both groups, to 0, 1, and 2; and in the third assessment, the OHI-S values were lower in the first group (which benefited from HBOT) compared to the control group. In Figure 4, the patient distribution based on OHI-S index, are presented at the first (a), second (b), and third (c) examination.
- Sulcus bleeding index (SBI): at their presentation, the patients of both groups had only values of 1, 2, 3, and 4 in SBI score, without value 0; in second clinical examination we found that SBI scores were significantly reduced in both study groups (HBOT and control), because of the same reasons (SRP treatment, correct teeth brushing); at the second and third determination, we found that the SBI scores decreased in the patients of both groups; and in third assessment, the SBI values were lower in the first group (HBOT) compared to the control group. In Figure 5, the patient distribution based on SBI index at first (a), second (b), and third (c) examination are depicted.
- Dental mobility (DM): We found that the DM has diminished in both groups; at the presentation all of patients had I-st and II-nd degree dental mobility; in the other two clinical examinations, we found that DM was reduced till physiological mobility, especially in patients who received HBO adjuvant therapy. Figure 6 depicts the patient distribution based on DM index at first (a), second (b), and third (c) examination.
- Pockets depth (PD): the measurements were effectuated with the periodontal probe and registered in the patients’ record; we found that the PD values were reduced in both groups of patients in the second and third clinical examination, but the reduction of PD was more significant in patients belonging to the HBOT group. In Figure 7, the patient distribution based on PD index at first (a), second (b), and third (c) examination is presented.

In conformity with the inclusion criteria, only patients with mild and moderate periodontal disease were admitted in study. Only two patients out of a total of 71 had a slight involvement of the furcation, therefore assessment of the involvement of furcation was not introduced in the study.

Table 5 present Chi-Square test *p*-values for test differences between control and test groups for all variables, at all three levels of investigation, with *p*-values. There are no significant differences, at any level, between the frequencies of the control and test groups, for any of the observed variables, no matter the treatment type.

In Table 6, Chi-Square test p-values are presented for test differences between groups for all variables, for test and control groups. The significance level is 0.1. Exceptions appear for the SBI index in the control group, which is significant at a 0.1 significance level, and all differences are significant at a 0.05 level. It can be noticed that there are significant differences in the effect of the three assessments, both in test or control groups.

At the finalisation of study, it was noted that, although patients did not initially mention their mild vertigo, mild tinnitus, and fatigability, at the end of HBO therapy, 21 patients (= 67.74%) stated, without being asked, that they no longer had mild vertigo, mild tinnitus symptoms and that they no longer present signs of chronic fatigue.

## 4. Discussion

The current standards of periodontal disease for the assessment of periodontitis is based primarily upon attachment and bone loss, and classifies the disease into four stages based on severity (I, II, III or IV) and three grades based on disease susceptibility (A, B or C). Therefore, is possible to create a staging and grading system for periodontitis [34].

Complete radiographic examination represents a part of the initial periodontal assessment for establishing the degree of horizontal and vertical alveolar bone loss. According to the 2017 World Workshop on the Classification of Periodontal and Peri-Implant Diseases and Conditions, a new periodontitis classification categorizes the disease based on a multi-dimensional staging and grading system [6,7]. Staging is determined by the severity of the disease at initial presentation and the complexity of disease management [35]. Wandawa et al. [36] applied 16 HBOT meetings after SRP, and their results were not significant from a statistical point of view. Soranta et al. [37] studied the action of HBOT on MMP-8 level in the saliva of chronic periodontitis patients. They applied 8 HBOT sessions, and observed that the results were meaningfully better than in monotherapy with SRP. The study of Robo et al. [38] and Chen et al. [39] proved that HBOT meaningfully decreases the anaerobic flora in subgingival sulcus. In their research, Balestra et al. [40] consider that studies in reference with HBOT should be extended, because this therapy can produce a strong stimulus at the level of the molecular reactions, but the requirements are in reference with “how much”, “how long”, and “how often” should this adjuvant therapy be used.

HBOT has a triggering role in bone remodelling, and the research effectuated by Lu et al. [41] demonstrated that the major impact of oxygen pressure is at the incipient phase of differentiation of osteoblasts. Salmón-González et al. [42] consider that there is a correlation between increasing oxygen pressure and increasing osteoblastic and osteoclastic activity. Studies of Huang et al. [43] mention that HBOT stimulates the fibroblast activity, the angiogenesis, and proceeds on leukocyte function for promoting lesion healing. In the article published by El-Baz et al. [44], it is highlighted that HBOT is a treatment that has become quite popular in the community of autistic patients and that this type of therapy has many benefits. In the research conducted by Bennett et al. [45], it is emphasized that there was some evidence of HBOT efficacy in the treatment of acute migraine, although study participants belonged to an unselected population. Devaraj et al. [46] believe that although HBOT has broad indications in various medical cases, the effective use of this type of dental treatment requires evidence, so research should also be undertaken in the field of dentistry to develop adjunctive therapy options with hyperbaric oxygen.

During the Tenth European Consensus Conference on Hyperbaric Medicine, recommendations for accepted and non-accepted clinical indications and practice of HBOT have been established [47]. In certain situations, it is necessary to assess the cost–benefit ratio, especially when patients pay full treatment or in case of presumably long treatments.

HBOT can be used as a monotherapy, or as a multimodal therapeutic variant. After a study published in 2017, Chhabra et al. [48] concluded, that ensuring the local hyperbaric oxygen atmosphere, the administration of growth factors, skin-substitutes, electric stimulation and local drainage, may constitute the conditions for local wound healing, a fact which may be applicable in the treatment of periodontal diseases. Marcinkowska et al. [49] consider that through properly addressing and evaluating methodological issues referring to HBOT, this therapy may have potential for the treatment of neuropsychological deficits in a wide range of neurological states, with importance in the treatment of trigeminal nerve affections.

HBOT as a preventive therapy may diminish the peril of dental implant failures in the maxillofacial area, including the irradiated patients [50,51]. The research of Hollander et al. [52], show that HBOT could be beneficial in nonirradiated patients with intraoral compromised wound healing. The healing of wounds after application of HBOT is underlined by Re et al. [21], Hollander et al. [52], and Shih et al. [53], especially in periodontal disease and oral submucous fibrosis. HBOT as adjuvant therapy in dentistry, associated with the other specific dental treatments, has benefits and facilitates the healing process, notwithstanding the potential complications that may appear [21,54].

According to the safety requirements of patients, HBO therapy should not be accepted to those with general medical conditions presented in exclusion criteria of patients. Adverse effects and complications that may arise during HBO therapy can be absolute, relative or potential [55,56]. The most common complications during HBO therapy are represented by middle and inner ear barotrauma, pulmonary barotrauma, sinus/paranasal and dental barotrauma, claustrophobia, and ophthalmological manifestations (as progressive myopia, new cataracts) [56,57,58]. Potential contraindications of HBOT are represented by the presence of pacemakers or any implantable devices, hereditary spherocytosis, pregnancy, hypoglycaemia, chronic obstructive pulmonary disease, allergic rhinitis, asthma, upper respiratory infections, and acute pulmonary edema. In defiance to its numerous uses, potential adverse effects of HBOT, which represent a possible hazard for patients, should be taken into account. All these impose that this knowledge referring to the complications and adverse effects of HBO therapy should be presented in the informed consent [59,60].

Medical conditions along with the comprehension of necessity in preservation of dental and gingival health represent the potential predictable of general and oral health status [61]. Diseases of the periodontal tissues also affect the tooth mobility degree [2,62]. The mobility of the teeth represents a utilized symptom in the evaluation of the health status of periodontal tissues, respectively, in obtaining success or failure of the periodontal treatment [35,63]. The extension of periodontal disease cannot be correctly estimated without the evaluation of the mobility degree [64]. The used clinical method for determining the tooth mobility is based on individual’s perception of tooth movement by application of a force on the tooth crown [65].

The acquaintances regarding the periodontal disease are significant for the prevention, and also for the preservation of the periodontal tissues health, in order to prevent severe subsequent disease. Incorrect oral hygiene induces the triggering of oral cavity tissue diseases, in particular of periodontal affections [5,66].

Future research directions will be correlated with the inclusion of a larger number of patients in clinical trial, longer duration of follow-up, thoroughgoing study of HBOT effects in severe periodontitis, respectively, by the enlargement of the researched items (such as patients which presents gingival recession, TMJ disorders).

The absence of assessments referring to severe periodontitis represents a limitation of this study. Another limitation of this clinical trial is represented by the relatively reduced number of patients in groups. The cost of HBO therapy is rather high, and represents another limitative reason. Additionally, because the price of HBO therapy in oral diseases is not discounted by the Romanian health care insurance system, the implementation of HBO therapy in real life presents difficulties.

## 5. Conclusions

Within the limits of the study, we concluded that the HBOT group of patients presented better values of OHI-S, SBI, DM and PD two months after the completion of HBO adjuvant therapy than patients in the second control group, but Chi-Square test *p*-values for test differences between groups, for all variables, at all the three levels of investigation, with *p*-values shown that there are no significant differences, at any level, between the frequencies of the control and test groups, for any of the observed variables, no matter the treatment type.

The majority of the patients of the first group declared that HBOT had beneficial effects on their general health status in symptoms prior to this adjuvant therapy, as tinnitus, vertigo, chronic fatigue, and migraines, but it is necessary to emphasise that all these are subjective statements.

Clinical trials with a greater the number of patients and longer follow-up time are required.

## Figures and Tables

**Table 1:** Sample patients.

	Group 1	Group 2
No of patients	31	40
Age (mean ± years)	48.5 ± 10.5	48.5 ± 10.5
Gender M/F	16/15	21/19
Confirmed periodontal disease	Mild to moderate	Mild to moderate

**Table 2:** Inclusion criteria of patients in study.

Inclusion Criteria
Male and female patients between 38 and 59 years of age
Non-smokers
Having at least 6 natural teeth on a half dental arch, excluding third molars
Periodontal symptomatology/confirmed diagnosis of moderate periodontal disease
Patient’s acceptance to participate in the study, with signed informed consent

**Table 3:** Exclusion criteria of patients in study.

Exclusion Criteria	
Smoker patients	Hereditary spherocytosis
Maximum scores of all studied periodontal indices	Fever
Periodontal treatment and antibiotic therapy in the last six months	Claustrophobia
Aggressive periodontitis	Convulsions
Endodontic affections	Cardiac simulators
Orthodontic patients	Implanted or external devices that control body functions
Infections	Pregnancy
Systemic disorders	Hospitalized patients
Upper respiratory and pulmonary disorders	Patients with mental disability
Cataract	Uncooperative patients
Eustachian tube dysfunction	Patients who refused to be included in the study

**Table 4:** Obtained results in the studied groups, according to the three clinical examinations.

Selected Patients (71)			Group I *HBOT* 31 Patients	Group II *Control* 40 Patients
First clinical examination, at patients presentation	Oral health index (OHI-S)	Score 0	-	-
		Score 1	3 (=9.67%)	5 (=12.50%)
		Score 2	12 (=38.40%)	17 (=42.50%)
		Score 3	16 (=51.61%)	18 (=45.00%)
	Sulcus bleeding index (SBI)	Score 0	-	-
		Score 1	2 (=6.45%)	3 (=7.50%)
		Score 2	5 (=16.12%)	7 (=17.50%)
		Score 3	17 (=54.83%)	21 (=52.50%)
		Score 4	7 (=22.58%)	9 (=22.50%)
	Dental mobility degree (DM)	0-Physiological mobility	-	-
		1st degree mobility	15 (=48.38%)	19 (=47.50%)
		2nd degree mobility	16 (=51.61%)	21 (=52.50%)
	Pockets depth (PD)	Normal	-	-
		Early/mild periodontitis	12 (=38.40%)	15 (=37.50%)
		Moderate periodontitis	19 (=61.29%)	25 (=62.50%)
Second clinical examination of both groups patients, at 1 week after the finalization of periodontal treatments	Oral health index (OHI-S)	Score 0	4 (=12.90%)	6 (=15.00%)
		Score 1	8 (=25.80%)	11 (=27.50%)
		Score 2	10 (=32.25%)	12 (=30.00%)
		Score 3	9 (=29.03%)	11 (=27.50%)
	Sulcus bleeding index (SBI)	Score 0	3 (=9.67%)	5 (=12.50%)
		Score 1	5 (=16.12%)	7 (=17.50%)
		Score 2	7 (=22.58%)	9 (=22.50%)
		Score 3	14 (=45.16%)	16 (=40.00%)
		Score 4	2 (=6.45%)	3 (=7.50%)
	Dental mobility degree (DM)	Physiological mobility	4 (=12.90%)	7 (=17.50%)
		1st degree mobility	16 (=51.61%)	19 (=47.50%)
		2nd degree mobility	11 (=35.48%)	14 (=35.00%)
	Pockets depth (PD)	Normal	3 (=9.67%)	5 (=12.50%)
		Early/mild periodontitis	17 (=54.83%)	19 (=47.50%)
		Moderate periodontitis	11 (=35.48%)	16 (=40.00%)
Third clinical examination of both groups patients, at 2 months after the finalization of HBOT in first group of patients	Oral health index (OHI-S)	Score 0	12 (=38.40%)	9 (=22.50%)
		Score 1	11 (=35.48%)	12 (=30.00%)
		Score 2	5 (=16.12%)	11 (=27.50%)
		Score 3	3 (=9.67%)	8 (=20.00%)
	Sulcus bleeding index (SBI)	Score 0	8 (=25.80%)	5 (=12.50%)
		Score 1	9 (=29.03%)	8 (=20.00%)
		Score 2	8 (=25.80%)	11 (=27.50%)
		Score 3	6 (=19.35%)	14 (=35.00%)
		Score 4	-	2 (=5.00%)
	Dental mobility degree (DM)	Physiological mobility	8 (=25.80%)	9 (=22.50%)
		1st degree mobility	17 (=54.83%)	20 (=50.00%)
		2nd degree mobility	6 (=19.35%)	11 (=27.50%)
	Pockets depth (PD)	Normal	9 (=29.03%)	8 (=20.00%)
		Early/mild periodontitis	16 (=51.61%)	18 (=45.00%)
		Moderate periodontitis	5 (=16.12%)	14 (=35.00%)

**Table 5:** Chi-Square test *p*-values for test differences between groups, for all variables, at all “three levels of investigation”.

Variable	*p*-Value	Variable	*p*-Value	Variable	*p*-Value	Variable	*p*-Value
OHI-S1	0.75	SBI1	0.99	DM1	0.65	PD1	0.91
OHI-S2	0.99	SBI2	0.91	DM2	0.82	PD2	0.81
OHI-S3	0.27	SBI3	0.21	DM3	0.66	PD3	0.28

**Table 6:** Chi-Square test *p*-values for test differences between both groups (SRP+HBOT and SRP) for all variables.

Variable	*p*-Value	
	Test	Control
OHI-S	0	0.016
SBI	0	0.078 *
DM	0.018	0.013
PD	0	0.026

* Significance level 0.1.

## Data Availability

Not applicable.
